# Season of conception and neurodevelopmental outcomes in singleton preterm infants less than 29 weeks gestation

**DOI:** 10.3389/fped.2025.1492429

**Published:** 2025-03-27

**Authors:** Smita Roychoudhury, Selphee Tang, Shabih U. Hasan, Kevin Fonseca, Abhay Lodha, Belal Alshaikh, Essa Alawad, Kamran Yusuf

**Affiliations:** ^1^ Department of Pediatrics, McMaster University, Hamilton, ON, Canada; ^2^ Alberta Health Services, Calgary, AB, Canada; ^3^ Department of Pediatrics, Section of Neonatology, Cumming School of Medicine, University of Calgary, Calgary, AB, Canada; ^4^ Department of Microbiology, Immunology and Infectious Diseases, University of Calgary and Alberta Precision Laboratories, Calgary, AB, Canada

**Keywords:** preterm infant, month of conception, winter months, neurodevelopmental outcome, Bayley III

## Abstract

**Background:**

Environmental factors vary with the seasons and affect fetal development. Our objective was to assess the impact of the season of conception on neurodevelopmental outcomes at 18–21 months corrected age in singleton infants <29 weeks’ gestation.

**Methods:**

A retrospective cohort study of infants born between 2006 and 2015 at a tertiary-level neonatal intensive care unit was conducted. The conception date was calculated as the date of birth minus gestational age plus 14 days, and the conception dates were then divided into winter and non-winter months. The primary outcomes were a composite score of <85 in any of the cognitive, language, or motor components of the Bayley Scales of Infant and Toddler Development, 3rd edition (Bayley-III), at 18–21 months corrected gestational age, and scores of <85 in the individual components. Multivariate logistic regression was used to assess confounders.

**Results:**

Of the 493 eligible infants, 162 (32.8%) were conceived in winter. There was no difference in the adjusted odds ratios (aORs) of any Bayley-III cognitive, language, or motor composite scores of <85 between the two groups. The aORs of cognitive and language scores <85 in the winter group were significantly higher [2.78, 95% confidence interval (CI) 1.37–5.65 and 1.97, 95% CI 1.07–3.62, respectively].

**Conclusion:**

Singleton infants <29 weeks’ gestation conceived in winter months have worse cognitive and language outcomes. Our results need validation in other and larger cohorts.

## Introduction

Fetal growth and development are dependent on intricate interactions between maternal, genetic, and environmental factors. While genetic and maternal factors may remain relatively constant throughout the year, environmental exposures may vary depending on the season of the year (1). The fetal period, especially the first trimester, is susceptible to the adverse effects of environmental perturbations. It is only in the last few decades that the profound effects of intrauterine exposures and events on extrauterine life are being appreciated (2). The term “exposome” describes the interaction of environmental factors that may interact with the genetic makeup of an individual, affecting outcomes (3–5). The neural exposome comprises non-genetic endogenous and exogenous factors that may affect brain development (6). The interaction between these potentially toxic factors and the brain has been called toxic stressor interplay (TSI) (6, 7). The brain is particularly vulnerable to environmental factors during fetal development, and there is a large body of literature to suggest that neurological disorders that manifest later in life have their origins during early fetal life, a critical period of brain development. Environmental factors that affect fetal development and vary with the seasons of the year include ambient temperature, sunlight, maternal diet, viral infections, and the use of pesticides (8, 9). These factors can potentially disrupt the maternal–placental–fetal (MPF) triad, leading to adverse outcomes (4, 7). Schizophrenia, a severe neuropsychiatric disorder, has been reported in several studies to be more common in people born during winter months and in people living at higher latitudes (10–12). Other mental health disorders that may have seasonality as a risk factor include mania, depression, bipolar disorder, and seasonal affective disorder (11). Although the data is not consistent, autism spectrum disorder (ASD) also has a seasonal prevalence with an inverse correlation between sunlight levels at the time of conception and ASD risk (13, 14). Congenital birth defects involving the nervous system, such as neural tube defects, are reported to be more common in infants conceived in late summer and early autumn (15, 16). Conception during winter months is associated with special educational needs and learning disorders in school children (1, 17, 18). In addition to neurological disorders having a seasonal prevalence, birthweight is also associated with season, with those born in winter having higher birthweight (19).

Despite the great strides made in the care of preterm infants, they continue to be at risk of neurodevelopmental impairment. Some of the risk factors associated with poor neurodevelopmental outcomes in preterm infants are well known and include gestational age, intraventricular hemorrhage, early and late onset sepsis, and bronchopulmonary dysplasia (20). It is, however, unknown if the season of conception has any effect on neurodevelopmental outcomes in preterm infants. Studying the effect of the season on patterns of disease can help in identifying possible risk factors that vary with the seasons (14, 21). Given the significant effect of season on brain development, the objective of our study was to investigate the association between the season of conception and neurodevelopmental outcomes at 18–21 months corrected gestational age in singleton preterm infants <29 weeks’ gestation. We hypothesized that infants conceived in winter months would have worse neurodevelopmental outcomes compared to infants conceived in non-winter months.

## Methods

### Study population

The neonatal intensive care unit (NICU) in Calgary maintains an electronic database of all admissions to the NICU. Standardized demographic, perinatal, and neonatal data are entered into the database by a research coordinator trained in database management. Infants <29 weeks’ gestation discharged from the NICU are followed by the regional Neonatal Follow-up Clinic, which serves the southern Alberta region. Singleton preterm infants <29 weeks’ gestation admitted to the NICU between 1 January 2006 and 31 May 2015 comprised the study population. Infants with major congenital malformations or chromosomal disorders were excluded. We also excluded infants of mothers with preeclampsia, gestational diabetes, and twins, as all these conditions have seasonal prevalence (22–25). The month of conception was calculated by subtracting the gestational age from the date of birth and adding 14 days (1). Based on the mean monthly temperatures in Calgary, November, December, January, and February were considered winter months, and the rest of the year was considered non-winter months (Supplementary Table 1). The Conjoint Health Research Ethics Board of the University of Calgary approved the study with a waiver of informed consent (Ethics ID REB 17-0153).

### Definitions

Perinatal and neonatal data were defined according to the Canadian Neonatal Network manual (26). Gestational age was assessed in the following order of preference: date of *in vitro* fertilization, first trimester ultrasonography, last menstrual period, obstetric estimate, and pediatric estimate. Antenatal steroid use was classified as any steroid use prior to birth. Data on maternal smoking were based on maternal self-reports and were defined as any cigarette smoking during pregnancy. Chorioamnionitis was defined as reported in maternal charts based on the presence of >1 of the following clinical signs: foul-smelling amniotic fluid, maternal fever during labor, uterine tenderness (without another cause), fetal tachycardia, and maternal leukocytosis. Small for gestation age (SGA) was defined as birthweight below the 10th percentile for gestational age and sex according to Canadian standards (27).

Intraventricular hemorrhage (IVH) was diagnosed based on the classification as described by Papile et al. (28) Periventricular leukomalacia was diagnosed on a cranial ultrasound performed at 3 weeks of age or later (29). Necrotizing enterocolitis (NEC) was diagnosed based on the criteria of Bell et al. (30) The diagnosis of retinopathy of prematurity (ROP) was based on the International Classification of ROP (31). Patent ductus arteriosus (PDA) presence was based on clinical and/or echocardiographic criteria. Confirmed sepsis was defined as the presence of a pathogenic organism in either blood or cerebrospinal fluid culture any time after birth.

### Outcomes

The primary outcomes were a composite score of <85 in any of the cognitive, language, or motor components of the Bayley Scales of Infant and Toddler Development, 3rd edition (Bayley-III) at 18–21 months corrected gestational age and scores of <85 in the individual components of Bayley-III. Secondary outcomes included death, scores of <70 in the individual components of Bayley-III, median scores of the three components of Bayley-III, cerebral palsy, and hearing and visual impairments. A cerebral palsy diagnosis was based on abnormal muscle tone and reflexes on physical and neurological examination and was classified according to the Gross Motor Function Classification System (GMFCS) (32) Mild cerebral palsy was defined as GMCFS levels 1 or 2, and moderate-severe cerebral palsy was defined as GMCFS levels 3, 4, or 5. Blindness was considered present if the infant had bilateral blindness with a corrected visual acuity of <20/200 in the better eye. Mild vision disability refers to those infants who had a corrected visual acuity <20/60 but >20/200 in the better eye; significant refractive errors, such as severe myopia or significant hypermetropia; or unilateral blindness. Deafness was defined as a bilateral sensorineural loss requiring amplification or cochlear implants. Mild hearing disability was defined as neurosensory hearing loss not requiring amplification or implants, or unilateral hearing loss requiring amplification.

### Data collection

Surviving infants <29 weeks’ gestation are followed prospectively at specific ages, including at 18–21 months corrected gestational age, at the Neonatal Follow-Up Clinic. These infants have comprehensive neurodevelopmental assessments by a multidisciplinary team comprising a neonatologist/developmental pediatrician, psychologist, physiotherapist, dietician, speech-language pathologist, nurse, social worker, ophthalmologist, and audiologist. The assessment includes historical, physical, and neurological examinations. At 18–21 months corrected age, the Bayley-III was administered by trained assessors to assess developmental functioning. The composite scores of the cognitive, language, and motor subtests were obtained. Caregiver sociodemographic information and education were obtained at the first follow-up visit. Members of the team were not aware that a study on the association of season of conception with neurodevelopmental outcome was being conducted in this population. The father’s occupation was classified according to the Blishen socioeconomic index for occupations in Canada (33).

### Statistics

Maternal and infant characteristics, the primary outcomes, and the secondary outcomes were compared for the winter and non-winter groups using the Pearson χ^2^ test or Fisher's exact test for categorical variables and Student’s *t-*test or the Mann–Whitney *U*-test for continuous variables as appropriate. Univariate and multivariable logistic regression analyses were applied for primary and secondary outcomes. For the multivariable analysis, the model was adjusted for gestation, sex, mode of delivery, small for gestational age, respiratory support at 36 weeks corrected gestational age, maternal education, IVH grades 3 or 4, ROP stage 3 or higher or requiring treatment, and confirmed sepsis. Adjusted odds ratios (aORs) and 95% confidence intervals (CIs) were calculated. The incidence of outcomes by month of conception, along with 95% confidence intervals, were examined graphically. All analyses were conducted using SAS 9.3 (SAS Institute Inc, Cary, North Carolina) with a significance level of 0.05.

## Results

**Figure 1:**
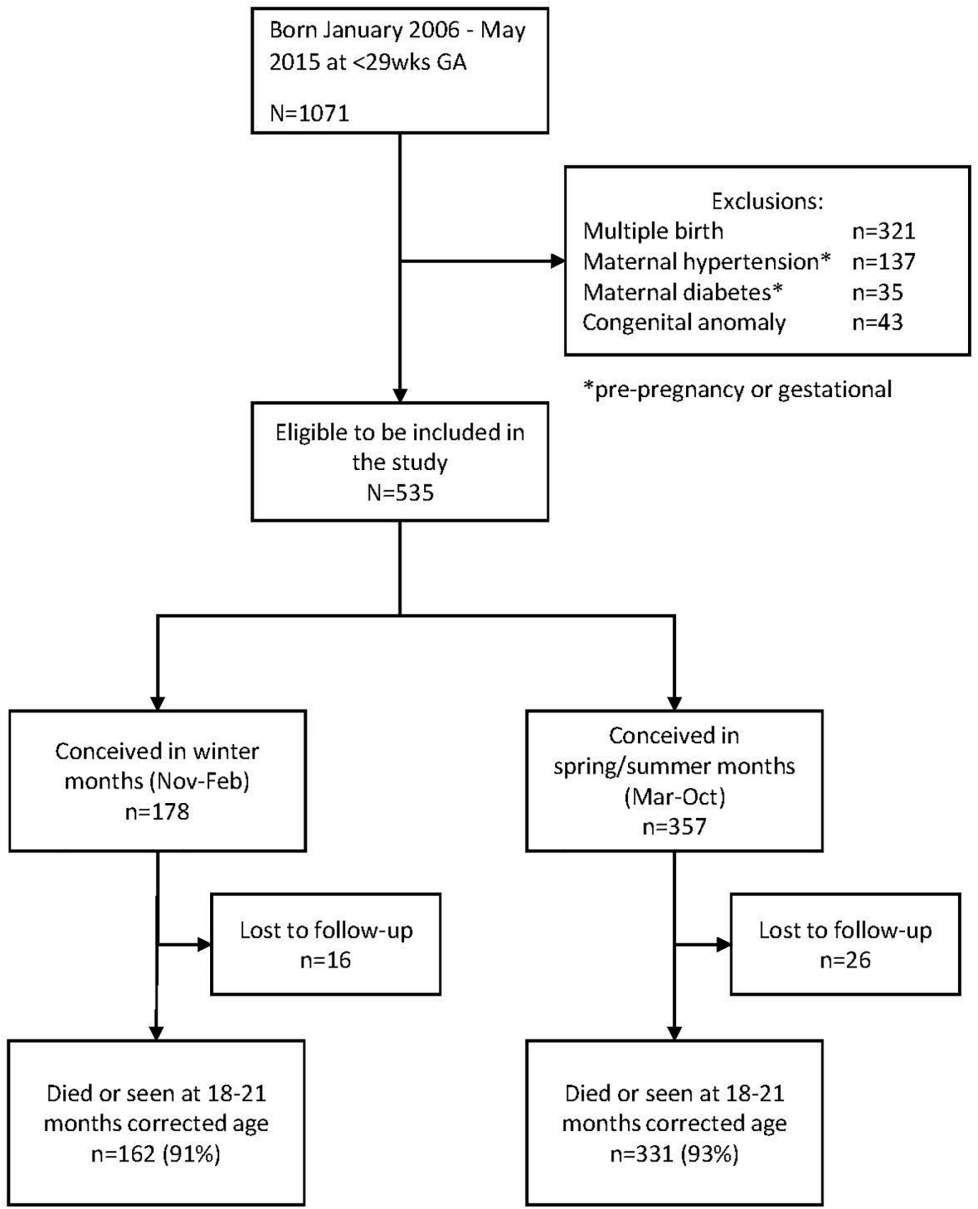
Flow diagram of the study population.

Figure 1 shows the flow diagram of the study. Of the 1,071 infants born during the study period, 536 met the exclusion criteria. Of the remaining 535 infants, 178 were conceived in the winter months and 357 in the non-winter months. In total, 16 infants in the winter group (9%) and 26 (7%) in the non-winter group were lost to follow-up, leaving 162 infants in the winter group and 331 in the non-winter group for analysis. There were 15 deaths in the winter group and 40 in the non-winter group.

### Maternal and neonatal characteristics

Table 1 shows the maternal characteristics of the two groups. Table 2 shows the neonatal characteristics. There was no difference between the two groups except for C-section rates, which were higher in the winter group. Neonates in the winter group had higher rates of respiratory support at 36 weeks, and there were more SGA neonates in the non-winter group.

**Table 1:** Maternal characteristics.

Characteristics	Winter *n* = 162	Spring/summer *n* = 331	*p*-value*
Maternal age at delivery, *n* (%)			0.871
<25 years	31 (19)	61 (18)	
25–34 years	98 (60)	208 (63)	
≥35 years	33 (20)	62 (19)	
Maternal age, years, mean (SD)	30 (6)	30 (6)	0.921
Multi gravida, *n*/*N* (%)	106/158 (67)	230/323 (71)	0.355
Ethnicity aboriginal/first nations, *n* (%)	4/115 (3)	11/247 (4)	0.665
Education high school or less, *n* (%)	44/136 (32)	96/274 (35)	0.590
Blishen score^a^, median (IQR)	41 (25)	45 (24)	0.442
	Missing = 42	Missing = 77	
Smoking during pregnancy, *n* (%)	43/160 (27)	72/327 (22)	0.236
Rupture of membranes ≥24 h, *n* (%)	50 (31)	102/326 (31)	0.924
Antenatal steroids, *n* (%)	133/161 (83)	276/328 (84)	0.666
Chorioamnionitis, *n* (%)	57/152 (38)	112/312 (36)	0.736
Maternal antibiotics, *n* (%)	106/160 (66)	237/328 (72)	0.173
C-section delivery, *n* (%)	83 (51)	136/329 (41)	0.038
Fertility treatment *n* (%)			0.38
None	152/160 (95)	311/326 (95)	
IUI or drug induced (clomid)	5/160 (50	5/326 (2)	
ART (IVF, ICSI)	3/160 (2)	10/325 (3)	

IQR, interquartile range; SD, standard deviation; IUI, intra-uterine insemination; ART, artificial reproductive technology; IVF, *in vitro* fertilization; ICSI, intracytoplasmic sperm injection.

Different variables have different denominators due to missing/unknown values.

* Chi-square test for categorical variables and *t*-test or the Mann–Whitney *U*-test for continuous variables.

^a^ Blishen score is based on the father's occupation.

**Table 2:** Neonatal characteristics.

Characteristics	Winter *n* = 162	Spring/summer *n* = 331	*p*-value*
Gestational age, weeks, mean (SD)	26.1 (1.5)	25.8 (1.5)	0.062
Birth weight, g, mean (SD)	921 (222)	895 (219)	0.222
Birth head circumference, cm, mean (SD)	24 (2)	24 (2)	0.269
	Missing = 2	Missing = 9	
Gestational age at discharge^a^, weeks, median (IQR)	38 (4)	38 (3)	0.871
	Missing = 2	Missing = 1	
Male, *n* (%)	90 (56)	179 (54)	0.757
Out born, *n* (%)	33 (20)	55 (17)	0.307
5-min Apgar score <7, *n* (%)	75/159 (47)	127/327 (39)	0.080
Cord pH <7.20, *n* (%)	24/118 (20)	45/253 (18)	0.556
Surfactant, *n* (%)	93 (57)	195/329 (59)	0.694
Caffeine, *n* (%)	151 (93)	309 (93)	0.952
Postnatal steroids (dexamethasone), *n* (%)	28 (17)	46/329 (14)	0.336
Days on ventilation, median (IQR)	38 (51)	35 (44)	0.988
	Missing = 4	Missing = 6	
Number of blood transfusions, median (IQR)	1 (3)	1 (4)	0.764
	Missing = 2	Missing = 8	
Respiratory support at 28 days of age, *n* (%)	126/151 (83)	259/322 (80)	0.433
Respiratory support at 36 weeks gestational age, *n* (%)	91/147 (62)	163/314 (52)	0.044
Total days of respiratory support, median (IQR)	76 (122)	67 (106)	0.131
	Missing = 22	Missing = 33	
Length of hospital stay^a^, median (IQR)	88 (38)	89 (34)	0.370
	Missing = 2	Missing = 1	
Presence of PDA, *n* (%)	101/160 (63)	217/330 (66)	0.567
PDA ligation, *n* (%)	32/160 (20)	53/330 (16)	0.280
SGA <10th percentile, *n* (%)	1 (1)	13 (4)	0.043
NEC, *n* (%)	18 (11)	55/329 (17)	0.101
IVH (any grade), *n* (%)	66 (41)	124 (37)	0.482
IVH grade III/IV, *n* (%)	28 (17)	61 (18)	0.756
ROP Stage 3 or more or treated ROP, *n* (%)	33/110 (30)	49/196 (25)	0.343
Periventricular leukomalacia, *n* (%)	9 (6)	17/329 (5)	0.857
Confirmed sepsis, *n* (%)	36/161 (22)	81/329 (25)	0.582

IQR, interquartile ratio; IVH, intraventricular hemorrhage; ROP, retinopathy of prematurity. Different variables have different denominators due to missing/unknown/not applicable values.

* Chi-square test for categorical variables, *t*-test, or Mann-Whitney *U*-test for continuous variables.

^a^ Excludes 40 in the spring/summer group and 15 in the winter group who died before discharge.

### Primary and secondary outcomes

Table 3 shows the univariate analysis of the primary and secondary outcomes. There was no difference in the primary composite outcome of any Bayley-III cognitive, language, or motor composite scores of <85. There was a greater proportion of infants with Bayley-III cognitive scores <85 and a greater proportion with language scores <70 in the winter group. Bayley-III cognitive and language median scores were significantly lower in the winter group. There was no difference in any of the other secondary outcomes.

**Table 3:** Neurodevelopmental outcomes at 18–21 months corrected age.

Outcomes	Winter *n* = 162	Spring/summer *n* = 331	*p*-value*
Primary outcomes			
Bayley-III cognitive, language, or motor composite <85^a^, *n* (%)	61/116 (53)	118/229 (52)	0.853
Bayley-III cognitive composite <85, *n* (%)	36/134 (27)	44/269 (16)	0.013
Bayley-III language composite <85, *n* (%)	48/117 (41)	80/232 (34)	0.231
Bayley-III motor composite <85, *n* (%)	29/122 (24)	58/236 (25)	0.866
Secondary outcomes			
Death, *n* (%)	15 (9)	40 (12)	0.349
Bayley-III cognitive composite <70, *n* (%)	8/134 (6)	11/269 (4)	0.401
Bayley-III cognitive composite, median (IQR)	90 (15)	95 (15)	0.045
Bayley-III language composite <70, *n* (%)	20/117 (17)	22/232 (9)	0.039
Bayley-III language composite, median (IQR)	86 (20)	91 (21)	0.011
Bayley-III motor composite <70, *n* (%)	14/122 (11)	20/236 (8)	0.359
Bayley-III motor composite, median (IQR)	94 (15)	92.5 (12)	0.642
Cerebral palsy, *n* (%)			0.531
None	134/146 (92)	272/291 (93)	
Mild	7/146 (5)	14/291 (5)	
Moderate/severe	5/146 (3)	5/291 (2)	
Visual impairment, *n* (%)			0.245
None	139/146 (95)	277/292 (95)	
Mild	7/146 (5)	10/292 (3)	
Bilateral blindness	0/146 (0)	5/292 (2)	
Hearing impairment, *n* (%)			0.565
None	140/143 (98)	279/286 (98)	
Mild	3/143 (2)	4/286 (1)	
Bilateral deafness	0/143 (0)	3/286 (1)	

* Chi-square or Fisher's exact test for categorical outcomes and the Mann–Whitney *U*-test for continuous outcomes.

^a^ Not all children seen at 21 months completed all components of the Bayley-III. If one or two of the cognitive, language, or motor composites were incomplete, but the completed score was <85, then it was included in the primary outcome. If one or two of the scores were incomplete but the completed score was ≥85, then the primary outcome was considered unknown.

Table 4 shows the results of the multivariate logistic regression models. There was no difference in the aORs of any Bayley-III cognitive, language, or motor composite scores of <85 between the two groups. However, the aORs of the cognitive scores and language scores <85 in the winter group were significantly higher (2.78, 95% CI 1.37–5.65, and 1.97, 95% CI 1.07–3.62, respectively). There was no statistically significant difference in the odds of any of the secondary outcomes between the two groups.

**Table 4:** Logistic regression models predicting outcomes at 18–21 months corrected age.

Outcomes	Crude OR (95% CI) for winter vs. spring/summer	Adjusted OR^a^ (95% CI) for winter vs. spring/summer
Primary outcome		
Bayley-III cognitive, language, or motor composite <85	1.04 (0.67–1.63)	1.14 (0.62–2.10)
Bayley-III cognitive composite <85	1.88 (1.14–3.10)	2.78 (1.37–5.65)
Bayley-III language composite <85	1.32 (0.84–2.09)	1.97 (1.07–3.62)
Bayley-III motor composite <85	0.96 (0.57–1.60)	0.65 (0.31–1.34)
Secondary outcomes		
Death	0.74 (0.40–1.39)	0.75 (0.37–1.49)
Bayley-III cognitive composite <70	1.49 (0.58–3.79)	2.74 (0.66–11.29)
Bayley-III language composite <70	1.97 (1.03–3.78)	2.26 (0.92–5.52)
Bayley-III motor composite <70	1.40 (0.68–2.88)	1.79 (0.55–5.75)
Cerebral palsy	1.28 (0.61–2.72)	1.31 (0.37–4.68)
Visual impairment or blindness	0.93 (0.37–2.33)	0.60 (0.16–2.27)
Hearing impairment or deafness	0.85 (0.22–3.35)	n/a^b^

^a^ Adjusted for gestational age, small for gestational age (SGA), C-section, respiratory support at 36 weeks, maternal education, male, severe intraventricular hemorrhage (IVH), severe retinopathy of prematurity (ROP), confirmed sepsis. For death, adjusted for gestational age, SGA, C-section, male, severe IVH, and confirmed sepsis (respiratory support at 36 weeks, maternal education, and severe ROP are excluded from the adjusted model as they are not available for a majority of those who died).

^b^ Model cannot be fit due to small numbers.

**Figure 2:**
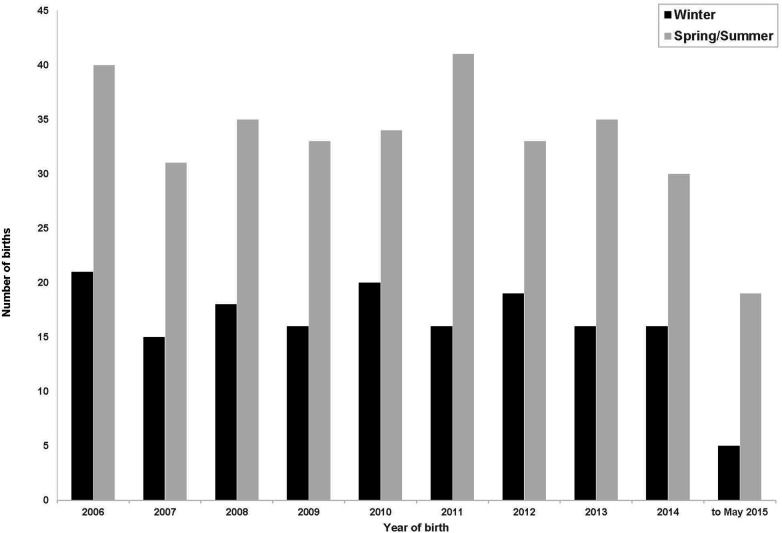
Births per year conceived in the winter and non-winter months.

Figure 2 shows the births per year conceived in winter and non-winter months each year during the study period.

**Figure 3:**
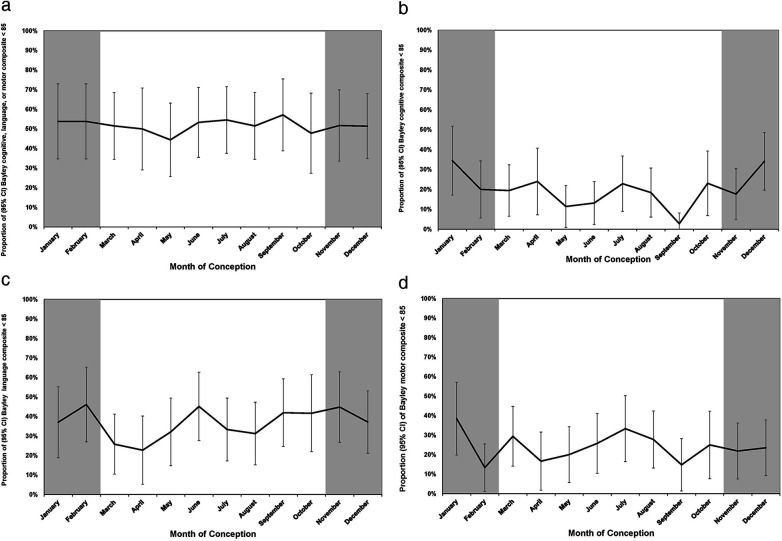
**(a)** Month of conception and proportion (95% CI) of Bayley cognitive, language, or motor composite scores <85 at 18–21 months. **(b)** Month of conception and proportion (95% CI) of Bayley cognitive composite scores <85 at 18–21 months. **(c)** Month of conception and proportion (95% CI) of Bayley language composite scores <85 at 18–21 months. **(d)** Month of conception and proportion (95% CI) of Bayley motor composite scores <85 at 18–21 months.

Figure 3 shows the incidence rates of Bayley-III scores <85 plotted by month of conception with 95% CIs. Figure 3a shows the month of conception and the proportion (95% CI) of Bayley composite of cognitive, language or motor scores of <85 at 18–21 months. The cognitive and language scores show a seasonal pattern with the highest rates in the winter months (Figures 3b,c). The language scores also showed a peak in June. There was no discernable seasonal pattern for the motor scores (Figure 3d) or for the composite outcome of the Bayley-III components. Table 5 shows the peak influenza activity in southern Alberta during the 2013–2014 to 2016–2017 seasons with the week of peak activity in winter.

**Table 5:** Weeks of peak flu activity in southern Alberta during flu seasons (1 September, week 36–31 May, week 23) from 2013 to 2017.

Resp season (1 Sept., wk. 36–31 May , wk. 23)	Total samples tested	PCR + ve flu A	PCR + ve flu B	PCR + ve flu A and flu B	Peak flu week activity	Max positivity^a^ (%)
2016–2017	34,249	3,970	776	4,746	1	28.9
2015–2016	30,250	4,166	1,724	5,890	6	34.7
2014–2015	30,396	4,236	958	5,194	52	43.1
2013–2014	29,375	3,948	564	4,512	52	38.1

PCR, polymerase chain reaction.

^a^ Maximum positivity for the respective flu week based on the number of samples tested.

## Discussion

Our data suggests that preterm infants <29 weeks’ gestation conceived in winter months have lower cognitive and language scores at 18–21 months corrected age as compared to infants conceived in non-winter months. To the best of our knowledge, this is the first study to report on neurodevelopmental outcomes in preterm infants based on the season of conception. Previous studies reporting on seasonal patterns of disease have used both the season of birth and conception. For our study, we chose the season of conception as the season of birth does not take into consideration critical periods of fetal development. Furthermore, the month of birth may be the same for infants of different gestational ages, but they will have different months of conception and different *in utero* exposures. Moreover, critical periods of development will be more constant from dates of conception, thus avoiding misclassification of exposure (8, 14, 34).

There are no studies on the season of conception and neurodevelopmental outcomes in preterm infants. However, previous studies have reported on developmental outcomes in schoolchildren based on the season of conception. Mackay et al., using a large cohort of schoolchildren from Scotland, demonstrated higher rates of learning difficulties, intellectual disability, and autistic spectrum disorder in children conceived in winter months with no effect on motor or physical impairment (1). These findings persisted after adjusting for potential confounders. Two large studies from California comprising more than 4.5 million children have reported a higher risk of autism and cerebral palsy in children conceived in winter (14, 21). Specific learning disabilities with lower standardized achievement scores in reading, mathematics, and science are described in schoolchildren born in the summer from June to August, corresponding to a winter season of conception (17). In a study from Canada, anencephaly was more common in infants conceived in January (35).

We observed a moderately higher risk of low cognitive and language Bayley-III scores in preterm infants conceived in winter months. However, season could be a proxy for season-dependent causal factors (36). By using the month of conception, a non-differential misclassification relative to the actual exposure may have been introduced into our cohort. Non-differential misclassification can underestimate the true association between an exposure and an outcome. Thus, if our findings are correct, the true causal factor may have a stronger association with lower Bayley-III scores than observed for the month of conception (14). Our findings persisted after adjusting for potential confounding factors with no effect on motor scores, suggesting the effect was specific and not the result of residual confounding. Our study was not designed to establish the mechanism of this association. There are two plausible exposures involved in the development of the nervous system that have a seasonal pattern and may be implicated in this association. These include maternal infection and maternal vitamin D levels.

The development of the fetal brain is a highly complex process under the influence of genetic and environmental factors, subject to a variety of adverse effects. Maternal infection, especially if it occurs early in pregnancy, can adversely affect neurodevelopment. Infants of mothers with serologically confirmed influenza A infection in early pregnancy were at higher risk of psychomotor delay at 6 months of age (37). Evidence from animal data suggests that maternal infection leads to both structural brain and behavioral changes in the offspring (38). In a mouse model, maternal influenza infection on day 9.5 of pregnancy, corresponding to the first trimester in humans, leads to abnormal corticogenesis and behavior in mouse pups reminiscent of autism (39). Rather than infection itself, maternal immune activation resulting from any cause can lead to brain injury and abnormal behavior in the progeny. Methods used to activate the maternal immune system in animal models include the administration of polyinosinic:polycytidylic (I:C), a double-stranded synthetic viral RNA, and bacterial lipopolysaccharide (LPS). A wide variety of brain abnormalities are described in rodent models of maternal immune activation following the administration of poly I: C. These include reduced brain volume, changes in the hippocampus,=abnormal dendritic spines, and migration of neurons in the offspring (38–40). Abnormal behaviors associated with these models include impaired learning and memory, communication deficits, impaired object recognition, and behavior suggestive of autism and schizophrenia (38, 40). Although still unclear, the mechanisms by which maternal immune activation leads to abnormal neurodevelopment in progeny may be mediated by cytokines, especially interleukin (IL)-6 and epigenetic mechanisms (38, 40, 41). Human data on the effects of maternal infection during pregnancy on the infant's neurodevelopment is, however, inconsistent (42). A negative association between maternal IL-6 levels and head circumference has been reported (43). Using magnetic resonance imaging, investigators have reported changes in the structure of the neonatal brain following antenatal maternal inflammation. Rasmussen et al. demonstrated an association between maternal IL-6 levels in the first trimester and abnormalities of the frontolimbic white matter in newborns. There was also an inverse association between IL-6 levels and cognitive function using Bayley-III at 1 year of age (44). Rudolph found a negative association between maternal IL-6 levels and working memory performance at 2 years of age and a changed pattern of whole-brain functional connectivity (45). Two other studies have also reported abnormalities of brain structure and behavior in the offspring of mothers with antenatal inflammation with elevated IL-6 levels (46, 47). In a study from Denmark, children born between 1980 and 2005 had a higher risk of autism if the mother had a viral infection in the first trimester (48). Fever during pregnancy is associated with autism and developmental delay in the offspring (49). The presence of auto-immune disorders in the mother, such as rheumatoid arthritis and celiac disease, is also associated with autism (50). Not all studies have, however, reported an association between maternal influenza infection and autism in the offspring (51). In Calgary, the peak weeks for influenza activity are in the winter months of December and January (Table 5), coinciding with the lower cognitive and language scores in our cohort.

Vitamin D is essential for normal fetal brain development and plays a role in brain and ventricular size, cell proliferation and differentiation, and growth factor signaling (52). Maternal vitamin D deficiency may lead to fetal vitamin D deficiency, as the fetus is entirely dependent on the mother for vitamin D (53). Importantly, sunlight is the major determinant of vitamin D, with marked seasonal variation as levels are lower in winter when there is less sunlight (54, 55). Maternal vitamin D insufficiency in the first trimester is associated with language impairment in the offspring at 5 and 10 years old (56). Higher vitamin D levels at 13.5 weeks of gestation are associated with higher mental and psychomotor scales of the Bayley Scales of Infant Development at 14 months old (57). In a study from Australia, umbilical cord blood levels of vitamin D were positively associated with language development in early childhood (58). Maternal vitamin D levels are also associated with attention deficit hyperactivity disorder (ADHD) in children, with lower levels a risk factor for ADHD (59). Vitamin D supplementation in early childhood is reported to lead to better cognition (60). A study from Sweden demonstrated that lower vitamin D levels estimated from dried blood spots after birth were associated with autism (61). Reduced sun exposure increases the risk for multiple sclerosis by affecting vitamin D levels (62). However, some studies have reported no association between neurodevelopment and maternal or umbilical cord blood levels of vitamin D. Keim et al. measured maternal or cord blood vitamin D levels at ≤26 weeks’ gestation and found no correlation with cognitive development, achievement, or behavior between 8 months and 7 years old (63). In Calgary, vitamin D insufficiency was reported in 80% of cord blood samples, which was related to the number of hours spent outdoors (64). In a study from Saskatchewan, which is at a similar latitude to Calgary, 70% of neonates born between December and February had vitamin D insufficiency (65). The 4 months with the lowest daylight hours in Calgary are November to February (Source: National Oceanographic and Atmospheric Administration, noaa.gov/weather, accessed on February 13, 2025). Both the cord blood vitamin D data from Calgary and the low daylight hours from December to February support our results of low cognition and language Bayley scores in babies conceived in this period.

Our results also show how the pregnancy exposome can change based on the season of conception and adversely affect the MPF triad, leading to poorer neurodevelopment outcomes for preterm infants. The placenta undergoes critical changes in the first trimester that are essential for normal fetal development. These include changing from histotrophic to hemotrophic nutrition and a three-fold increase in the intraplacental oxygen (66). Vitamin D is essential for normal placental development, and from early pregnancy, the human placenta expresses all the apparatuses for vitamin D signaling. Overall, vitamin D is involved in embryo implantation and decidual development, supports normal pregnancy and fetal growth, regulates multiple placental hormones, and attenuates the maternal and placental response to infection (67–69). Vitamin D deficiency in early pregnancy can adversely affect placental development and function, leading to a compromised fetus. The importance of vitamin D in fetal brain development, with low vitamin D altering the neural exposome, has been discussed above. We have also discussed how an altered pregnancy and neural exposome due to maternal infection or maternal auto-immune disease could explain our results. Maternal infection in early pregnancy can also change the MPF triad, impact the development of the placenta, and affect fetal growth and development (70).

Our study has several strengths. In Canada, there is universal healthcare, and almost all mothers have a first trimester ultrasound, giving confidence to the accuracy of the estimation of the month of conception in our cohort. Misclassification of the birth of conception can lead to erroneous results (14). We also used the month of conception rather than the month of birth as this gives a more accurate time of exposure to environmental insults at critical times of development. Our study includes all infants in the cohort rather than matched controls from the cohort as some studies on the seasonal effects on disease have done. This lessens the chance of bias in our cohort (71). Importantly, the inclusion of multiple years of conception allowed us to recognize long-term seasonal trends that did not change from year to year, giving us confidence that the seasonal trends were not by chance (13). There are also limitations to our study. Our cohort was from a single center, making generalization of our results difficult. We also did not measure vitamin D levels or assess evidence of maternal inflammatory activation in the mothers of our cohort. Further studies will be required to answer this question. However, based on data from Calgary on the seasonal prevalence of influenza, umbilical cord levels of vitamin D, and daylight hours, we do provide plausible biological explanations for our results.

In summary, over the last decades, there has been a concerted effort to understand the effect of seasonal environmental exposures during pregnancy that may affect fetal development, leading to long-term physical, developmental, and mental health problems. Our study provides evidence of how potential seasonal environmental exposures change the pregnancy and neural exposome and adversely affect the neurodevelopmental outcomes of preterm infants. It is important to recognize these TSIs, especially during critical periods of development such as early pregnancy, as they allow a better understanding of environment and gene interactions. This may suggest not only possible mechanisms of disease but also possible interventions to improve outcomes (72). The results of our study need to be confirmed by larger multi-center studies.

## Data Availability

The datasets presented in this article are not readily available because the data generated for the current study are not publicly available due to data transfer agreements and approvals that specifically indicate that the data will not be distributed outside the Alberta Health Services. The datasets used for the current study are available from the corresponding author on reasonable request. Requests to access the datasets should be directed to kyusuf@ucalgary.ca.
