# Supplementary material for: Season of conception and neurodevelopmental outcomes in singleton preterm infants less than 29 weeks gestation
Source: Front Pediatr. 2025 Mar 27;13:1492429. doi: 10.3389/fped.2025.1492429 (PMC11983454; doi:10.3389/fped.2025.1492429)
Supplement: Supplementary file 1 [file Table1.docx]

Supplementary Table 1

30-Year Temperature Averages for Calgary, 1981 to 2010

| High °F | Low °F |  | High °C | Low °C |
| --- | --- | --- | --- | --- |
| 30 | 8 | January | -1 | -13 |
| 33 | 11 | February | 1 | -11 |
| 40 | 18 | March | 4 | -8 |
| 52 | 28 | April | 11 | -2 |
| 61 | 38 | May | 16 | 3 |
| 68 | 45 | June | 20 | 7 |
| 74 | 50 | July | 23 | 10 |
| 73 | 48 | August | 23 | 9 |
| 64 | 39 | September | 18 | 4 |
| 53 | 29 | October | 12 | -1 |
| 38 | 17 | November | 3 | -8 |
| 30 | 9 | December | -1 | -13 |

10-year temperature averages for Calgary, 2010 to 2019

| High °F | Low °F |  | High °C | Low °C |
| --- | --- | --- | --- | --- |
| 32 | 12 | January | 0 | -11 |
| 29 | 9 | February | -2 | -13 |
| 39 | 19 | March | 4 | -7 |
| 51 | 29 | April | 10 | -2 |
| 62 | 39 | May | 17 | 4 |
| 69 | 47 | June | 20 | 8 |
| 75 | 52 | July | 24 | 11 |
| 74 | 50 | August | 23 | 10 |
| 64 | 42 | September | 18 | 5 |
| 53 | 31 | October | 11 | -1 |
| 38 | 18 | November | 3 | -8 |
| 30 | 11 | December | -1 | -12 |

[Calgary AB Average Temperatures by Month - Current Results](https://www.currentresults.com/Weather/Canada/Alberta/Places/calgary-temperatures-by-month-average.php)

<https://www.currentresults.com/Weather/Canada/Alberta/Places/calgary-temperatures-by-month-average.php> (Accessed on September 5, 2024)
